# Molecular Mechanism of Local Drug Delivery with Paclitaxel-Eluting Membranes in Biliary and Pancreatic Cancer: New Application for an Old Drug

**DOI:** 10.1155/2015/568981

**Published:** 2015-04-23

**Authors:** Sookhee Bang, Sung Ill Jang, Su Yeon Lee, Yi-Yong Baek, Jieun Yun, Soo Jin Oh, Chang Woo Lee, Eun Ae Jo, Kun Na, Sugeun Yang, Don Haeng Lee, Dong Ki Lee

**Affiliations:** ^1^Department of Internal Medicine, Gangnam Severance Hospital, Yonsei University College of Medicine, 712 Eonjuro, Gangnam-gu, Seoul 135-720, Republic of Korea; ^2^Department of Internal Medicine, Kangnam Sacred Heart Hospital, Hallym University College of Medicine, Seoul, Republic of Korea; ^3^Department of Bioevaluation Center, Korea Research Institute of Bioscience and Biotechnology, Cheongwon, Republic of Korea; ^4^Department of Research and Development, Taewoong Medical Co., Gimpo, Republic of Korea; ^5^Department of Biotechnology, The Catholic University, Bucheon, Republic of Korea; ^6^Department of New Drug Development, School of Medicine, Inha University, Incheon, Republic of Korea; ^7^Department of Internal Medicine, School of Medicine, Inha University, Incheon, Republic of Korea

## Abstract

Implantation of self-expanding metal stents (SEMS) is palliation for patients suffering from inoperable malignant obstructions associated with biliary and pancreatic cancers. Chemotherapeutic agent-eluting stents have been developed because SEMS are susceptible to occlusion by tumor in-growth. We reported recently that paclitaxel-eluting SEMS provide enhanced local drug delivery in an animal model. However, little is known about the molecular mechanisms by which paclitaxel-eluting stents attenuate tumor growth. We investigated the signal transduction pathways underlying the antiproliferative effects of a paclitaxel-eluting membrane (PEM) implanted in pancreatic/cholangiocarcinoma tumor bearing nude mice. Molecular and cellular alterations were analyzed in the PEM-implanted pancreatic/cholangiocarcinoma xenograft tumors by Western blot, immunoprecipitation, and immunofluorescence. The quantities of paclitaxel released into the tumor and plasma were determined by liquid chromatography-tandem mass spectroscopy. Paclitaxel from the PEM and its diffusion into the tumor inhibited angiogenesis, which involved suppression of mammalian target of rapamycin (mTOR) through regulation of hypoxia inducible factor (HIF-1) and increased apoptosis. Moreover, implantation of the PEM inhibited tumor-stromal interaction-related expression of proteins such as CD44, SPARC, matrix metalloproteinase-2, and vimentin. Local delivery of paclitaxel from a PEM inhibited growth of pancreatic/cholangiocarcinoma tumors in nude mice by suppressing angiogenesis via the mTOR and inducing apoptosis signal pathway.

## 1. Introduction

Malignant biliary obstruction is associated with biliary cancer, pancreatic cancer, and other local cancers. Endoscopic biliary drainage with self-expanding metal stents (SEMS) is the treatment of choice for palliation in patients with an unresectable biliary obstruction [1, 2]. A metallic stent covered with a paclitaxel-incorporated membrane (MSCPM) has been developed to promote the antitumor effect against extrahepatic cholangiocarcinoma, spreading along the bile duct wall, and to sustain stent patency by inhibiting tumor in-growth into the SEMS [3–7]. A double-layered MSCPM has been developed, which has a bile resistant inner layer of polytetrafluoroethylene and an outer layer of drug-containing polyurethane with pluronic F-127, a surfactant for effective drug delivery. We have reported that paclitaxel-eluting stents with 10% pluronic F-127 (MSCPM-II; Taewoong Medical Co., Gimpo, Korea) are safe and provide enhanced local drug delivery (LDD) in an animal model [8]. MSCPM-II is currently awaiting human application.

The chemotherapeutic mechanism of paclitaxel is to stabilize microtubules during mitosis and to arrest cell growth [9, 10]. In addition, paclitaxel has antiangiogenic and antimetastatic properties [11, 12]. The clinical application of paclitaxel in cancer treatment is considerably limited due to its poor availability from systemic administration [13]. Therefore, many efforts have been made to develop an alternative paclitaxel delivery system to increase its availability at tumor sites and to maximize therapeutic efficacy while minimizing side effects [14]. Furthermore, paclitaxel is useful for locoregional cancer therapy because it has good pharmacokinetic characteristics (e.g., lipophilic and rapid cellular uptake) [15]. Paclitaxel-eluting covered metal stents, which were introduced recently, may prevent occlusion from tumor in-growth due to the antitumor effect of paclitaxel. The diverse molecular signaling pathways generated by paclitaxel-eluting stents that exert antiproliferative, proapoptotic, and antiangiogenic effects in tumors have not been identified.

In the present study, we report a number of molecular pathways and cellular mechanisms that are associated with subtumoral implantation of a paclitaxel-eluting membrane (PEM), which is of identical composition to the outer layer of MSCPM-II that inhibits tumor growth. We analyzed the protein profile by immunoblot/immunoprecipitation analyses and validated the profile by immunofluorescence in pancreatic and cholangiocarcinoma xenograft tumors. We then explored the antiproliferative/apoptotic/antiangiogenic effects of the PEM, a clinically relevant drug-eluting stent identified in our study, to reveal its potential therapeutic significance for inoperable malignant biliary obstructions.

## 2. Materials and Methods

### 2.1. Cell Lines and Antibodies

The human pancreatic cancer cell lines PANC-1 and CFPAC-1 were cultured in Dulbecco's modified Eagle's medium and the cholangiocarcinoma cell lines HuCCT-1 and SCK were cultured in RPMI-1640. PANC-1 and CFPAC-1 cells were purchased from the ATCC (Manassas, VA, USA). HuCCT-1 and SCK cells were procured from the Health Science Research Resources Bank (Osaka, Japan) and Dr. Dae-Ghon Kim of Chonbuk National University Medical School and Hospital (Jeonju, Korea), respectively. All cell lines were maintained in a humidified incubator at 37°C with 5% CO_2_. Antibodies against S6K, phospho-S6K, S6, phospho-S6, 4EBP1, phospho-4EBP1, cleaved caspase-3, CHOP, Bax, Bim, BCl-2, cyclin B1, HIF-1*β*, CD44, SPARC, vimentin, and GAPDH were obtained from Cell Signaling Technology. CD-31 and VEGF were purchased from Abcam (Cambridge, MA, USA). HIF-1*α*, VEFGR2/Flk-1, and MMP-2 were obtained from Santa Cruz Biotechnology.

### 2.2. Tumor Xenograft and Treatment

Female 6–8-week-old athymic nude mice were purchased from Orient Bio (Kyunggido, Korea) for subcutaneous xenografts. To establish the tumor xenograft model, 2 × 10^6^ cells were suspended in 200 *μ*L plain growth media (DMEM or RPMI-1640) and injected subcutaneously into spaces under the dorsal skin. Tumors were measured every other day using calipers, and their volumes were calculated by the following formula: 0.5 × length × width^2^. The animal's body weight was monitored every other day. When tumor volume reached 100 mm^3^, the mice were anesthetized with a mixture of Zoletil (30 mg/kg) and Rompun (10 mg/kg) i.p., and the PEMs were surgically implanted underneath the tumors. All animal studies were conducted in compliance with the policy of the animal care and use committee of the Korean Research Institute of Bioscience and Biotechnology.

### 2.3. Preparation of Paclitaxel-Eluting Membrane

PEMs were fabricated using a mold. Briefly, 400 mg of polymer was dissolved in 10 mL of tetrahydrofuran (THF). Pluronic F-127 (Plu; 40 mg) and 1–10 wt% paclitaxel (PTX, 4–40 mg) were dissolved in THF and then added to the PU/THF solution and mixed by vortex and sonication. A 200 *μ*L aliquot of the mixture was poured into a dish-shape Teflon mold. The air-dried paclitaxel-eluting dish-shaped membranes were carefully peeled off the Teflon mold. Additionally, the PU membrane without paclitaxel and Plu (control) or Plu alone (control + Plu) was fabricated by the same method as the* in vivo* control.

### 2.4. Immunoprecipitation and Immunoblot Analyses

Tumors were minced coarsely and homogenized with lysis buffer containing 100 mM Tris (pH 7.4), 150 mM NaCl, 1% Triton X-100, 15% glycerol, 1 mM PMSF, phosphatase inhibitor mixtures 2 and 3 (Sigma, St. Louis, MO, USA), and a protease inhibitor mixture (Sigma). The homogenates were centrifuged at 14,000 rpm and 4°C, and the supernatants were used. Protein concentration was estimated using the Bio-Rad protein assay (Bio-Rad, Munich, Germany). For immunoprecipitation, tumor lysate protein (1 mg) was incubated with 2 *μ*g of anti-Bax or anti-Raptor antibodies (Cell Signaling Technology, Danvers, MA, USA) as indicated for 16 h at 4°C and precipitated with 50 *μ*L of TrueBlot anti-rabbit Ig IP beads (eBioscience, San Diego, CA, USA) for an additional 3 h. Then, the samples were washed five times with lysis buffer, and SDS-loading sample buffer was added. Immunoprecipitates and total tumor lysates (30 *μ*g) were separated on NuPage 4–12% gradient Bis-Tris gels (Invitrogen, Grand Island, NY, USA). Following sodium dodecyl sulfate-polyacrylamide gel electrophoresis, the proteins were transferred to nitrocellulose membranes (Bio-Rad). The blots were blocked in TBS with 0.1% Tween-20 buffer (TBST) containing 5% nonfat dry milk for 1 h at room temperature. The membrane was incubated overnight at 4°C with one of the following primary antibodies. After repeated washings with TBST, the membranes were incubated with goat anti-mouse IgG-horse radish peroxidase (HRP) or goat anti-rabbit IgG-HRP (Santa Cruz Biotechnology, Santa Cruz, CA, USA) for 1 h at room temperature before washing again with TBST. TrueBlot anti-rabbit Ig HRP-conjugated secondary antibody (eBioscience) was used to detect coimmunoprecipitates of Bim or hypoxia-inducible factor (HIF)-1*α* blots with a chemiluminescence reagent (Bio-Rad). GAPDH expression levels were used to normalize protein loading. The sizes of the molecular weight markers (in kilodaltons) are indicated on the left. All critical blots and immunoprecipitation experiments were repeated at least three times.

### 2.5. Immunofluorescence from Tumor Samples

Tumors were immersed in OCT compound (Leica Biosystems, Richmond, CA, USA) and frozen in liquid nitrogen. The sections (5 *μ*m) were permeabilized, blocked with 10% goat antiserum and 0.25% Triton X-100 in PBS to avoid nonspecific binding for 60 min, and subsequently incubated with rabbit anti-cleaved caspase-3 (1 : 400), rabbit anti-CD31 (1 : 400), or mouse monoclonal anti-vascular endothelial growth factor receptor-2 (VEGFR2) (1 : 400) antibodies. Next, the sections were washed and further incubated with the corresponding Alexa-488-conjugated secondary antibodies (Invitrogen). Nuclei were stained with DAPI (Invitrogen). Fluorescence images were acquired using an Axiovert 135 microscope (Carl Zeiss, Thornwood, NY, USA).

### 2.6. Immunohistochemistry

The tumors were removed and fixed in 10% phosphate-buffered formaldehyde at room temperature until sectioning. Briefly, all tumors were serially sectioned and tissue sections (5 *μ*m thick) obtained from the paraffin blocks were stained with hematoxylin and eosin (H&E) using standard histological techniques.

### 2.7. Measurement of Paclitaxel Content in Tumor Tissue by Liquid Chromatography-Tandem Mass Spectrometry (LC-MS/MS)

Tumors and plasma samples were prepared and paclitaxel concentrations were analyzed by LC-MS/MS. Briefly, the samples were extracted with 100% acetonitrile containing carbamazepine as an internal standard, and chromatography was conducted on an Xterra C18 column (50 × 2.1 mm i.d., 5 *μ*m, Waters, Milford, MA, USA) with a SecurityGuard C_18_ guard column (2.0 × 4.0 mm i.d., Phenomenex, Torrance, CA, USA) maintained at room temperature. The mobile phase was 95% v/v solvent A (deionized water containing 0.1% v/v formic acid)/5% v/v solvent B (acetonitrile containing 0.1% v/v formic acid) at a flow rate of 0.4 mL/min. A linear gradient of the two solvents was used: start at 95% A and hold for 0.5 min, ramp to 5% A to 0.6 min, and hold until 4 min. The flow rate was 0.4 mL/min throughout the gradient. The retention times of paclitaxel and the internal standard (IS) were 3.0 and 2.8 min, respectively. The electrospray ionization source was operated at 5500 V and 550°C. The samples were analyzed via multiple reaction monitoring. The monitoring ions were set as* m/z* 876 → 308 for paclitaxel and* m/z* 237 → 194 for the IS. The scan dwell time was 0.1 sec for each channel. Acquisition and analysis of data were performed using the Analyst software ver. 1.5.2 (Applied Biosystems, Foster City, CA, USA).

### 2.8. Statistical Analysis

Data are presented as means ± standard deviations. The statistical analysis was performed using Student's* t*-test when appropriate. Significance was established when *P* < .05. All experiments were performed a minimum of three times.

## 3. Results

### 3.1. The PEM Reduces Growth of Pancreatic/Biliary Xenograft Tumors in Nude Mice via Induction of Apoptosis and Endoplasmic Reticulum- (ER-) Stress in Tumors

**Figure 1:**
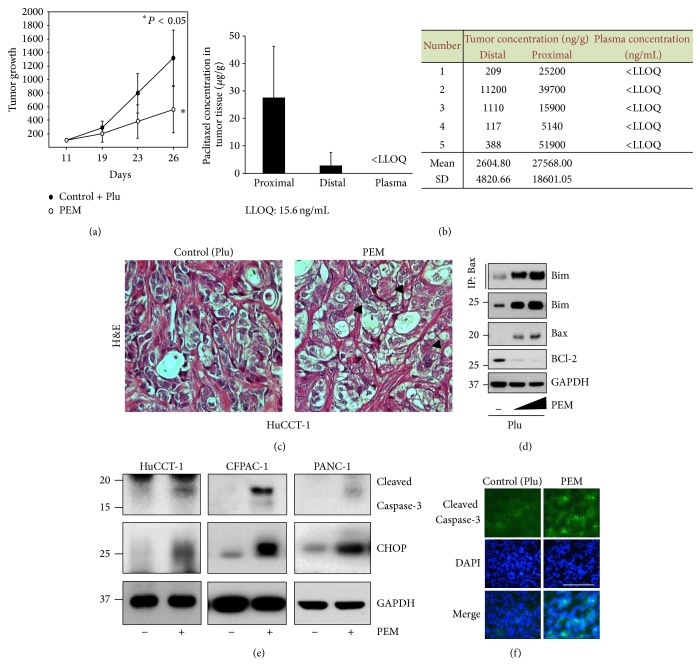
The paclitaxel-eluting membrane (PEM) reduces tumor growth by inducing apoptosis and endoplasmic reticulum- (ER-) stress in xenografted tumors. (a) The PEM (PTX 10% + Plu) was implanted once the SCK tumor reached 100 mm^3^. Data are presented as means ± standard deviation (SD). Tumor size was measured at the indicated time points after PEM implantation. (b) Plasma and tumor concentrations of paclitaxel in SCK tumor-bearing nude mice following PEM implantation for 15 days. Paclitaxel levels in plasma and tumor sections were determined by LC-MS/MS. Each data point represents the average of five mice ± SD. P, proximal to the PEM; D, distal to the PEM; ∗, lowest limit of quantification (LLOQ: 15.6 ng/mL). Raw data are shown in the inset. (c) Representative H&E stained PEM-implanted HuCCT-1 tumor. Arrowhead, necrotic tumor cells. (d) The PEM induced apoptosis in CFPAC-1 tumors. CFPAC-1 xenograft tumors were treated with 0, 1, and 5% PEM and implanted for 7 days. BCl-2, Bim, and Bax expression were detected in tumor lysates by Western blotting. Bax and Bim were immunoprecipitated (IP) with Bax. (e) The PEM induced apoptosis and ER-stress in HuCCT-1, CFPAC-1, and PANC-1 tumors. Cleaved caspase-3 and CHOP were detected in tumor lysates by Western blotting. (f) Immunofluorescence staining of cleaved caspase-3 in SCK tumor tissue section from tumor treated with the PEM or control (+Plu). Green, cleaved caspase-3 and blue, nuclei stained with DAPI. Original magnification, ×40. Scale bar: 50 *μ*m.

We have demonstrated previously that paclitaxel-eluting SEMS containing 10% pluronic acid F-127 (MSCPM-II) provide enhanced LDD to the porcine bile duct [8]. Hence, we examined the molecular mechanism underlying the antiproliferative effects of PEM implantation in tumor-bearing nude mice. Xenografted nude mice were implanted with pluronic acid alone (control + Plu), 5% and 10% paclitaxel and pluronic acid (PTX + Plu)-eluting membrane (PEM), or a bare membrane (control) for 15–20 days. First, we determined whether PEM implantation caused tumor shrinkage. We observed that the PEM was clearly effective for reducing tumor growth, compared with the control (bare membrane or Plu only) (Figure 1(a), Supplementary Figures S1 and S2 in supplementary material available online at http://dx.doi.org/10.1155/2015/568981). Moreover, no host toxicity was observed (data not shown). The drug-release study showed a 10-fold greater concentration of paclitaxel in tumors proximal to the PEM than in those distal to the PEM 15 days after implantation, whereas plasma concentrations of paclitaxel following membrane implantation remained less than the lower limit of quantitation (Figure 1(b)). Paclitaxel easily penetrated the cells of the tumor mass due to its lipophilic properties, which led to chronic retention of the drug in the tumor tissue. This result indicates that local delivery of PTX to tumor tissue is effective for reducing tumor growth without detectable systemic levels (Supplementary Figure S3). The significance of PEM-induced inhibition of tumor growth and whether it is related to cellular apoptosis or necrosis should be further investigated. The H&E histological analysis showed increased necrosis in the PEM-implanted tumors compared with the control (Figure 1(c)). We examined the effect of the PEM on expression of apoptosis-related proteins in tumor lysates to explore the link between tumor regression and induction of apoptosis by the PEM. Because the Bcl-2 family protein members are major regulators of the mitochondrial apoptotic pathway, we examined the expression of Bcl-2 family proteins in response to PEM implantation into tumors. As shown in Figure 1(d), expression of the proapoptotic proteins Bax [16] and Bim [17] increased in tumors implanted with the PEM. In contrast, the PEM induced downregulation of Bcl-2 expression (Figure 1(d)). It has been demonstrated that Bim initiates activation of Bax through a direct interaction* in vitro* [16]. To confirm the* in vivo* interaction between Bax and Bim, tumor lysates from either control or PEM-treated CFPAC-1 xenograft tumors were subjected to immunoprecipitation with Bax antibody, and the bound Bim was detected by immunoblotting. We found a dose-dependent increase in Bax/Bim binding in the PEM-implanted tumor lysates (Figure 1(d)). Western blotting revealed that the PEM induced expression of cleaved caspase-3, a characteristic of apoptosis, in xenografted tumors (Figure 1(e)). The immunofluorescence analysis confirmed that the PEM increased cleaved caspase-3 expression in tumors (Figure 1(f)). Because paclitaxel induces mitochondrial apoptosis and excess ER stress [18], we examine whether ER stress was involved in the PEM induced inhibition of tumor growth. Although the complete mechanism associated with ER stress-mediated apoptosis is unclear, downstream ER stress signaling could be correlated with activation of the c/EBP homology protein (CCAAT-enhancer-binding protein homologous protein [CHOP], an ER stress marker) [19]. As shown in Figure 1(e), expression levels of CHOP increased markedly in tumors implanted with the PEM. These results indicate that the PEM also inhibited tumor growth via induction of ER stress in addition to apoptosis.

### 3.2. The PEM Reduces Growth of Xenografted Tumors in Nude Mice by Suppressing mTORC1 Signaling and HIF-1 Regulation

**Figure 2:**
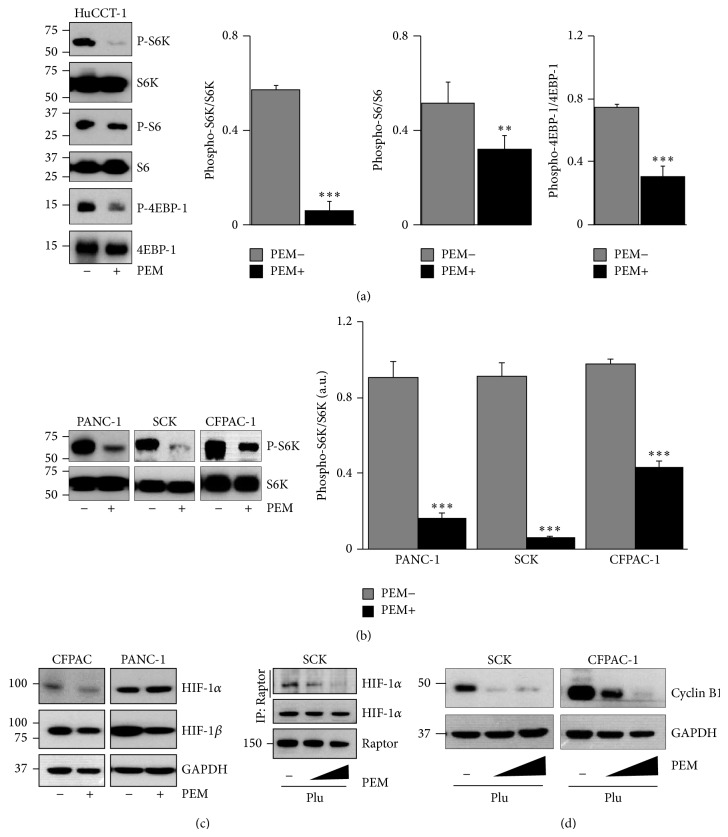
Paclitaxel-eluting membrane (PEM) implantation inhibits mTORC1 activation and hypoxia-inducible factor (HIF)-1 regulation in xenografted tumors. (a) Western blotting of phospho-S6K, S6K, phospho-S6, S6, phospho-4EBP1, and 4EBP1 expression levels in tumor lysates from HuCCT-1 tumors implanted with the PEM or control (+Plu) (left). Relative quantification of phosphoprotein expression levels is shown in the control (gray bars) or PEM (black bars) implanted tumors. Values are corrected for corresponding total antibody protein ^∗∗^ *P* < 0.01; ^∗∗∗^ *P* < 0.001 (right). (b) Western blotting of phospho-S6K and S6K expression levels in tumor lysates from indicated tumors from the PEM-implanted or control groups (left) and relative quantification of phospho-S6K expression levels ^∗∗∗^ *P* < 0.001 (right). (c) Western blot of HIF-1*α* and HIF-1*β* in a protein extract from indicated tumors implanted with the PEM or control for 20 days (left). SCK xenograft tumors were treated with 0, 5, and 10% PEM and implanted for 7 days. Tumor protein lysates were prepared and analyzed for Raptor and HIF-1*α* protein expression. Immunoprecipitation and Western blotting were used to detect the endogenous Raptor-HIF-1*α* interaction (right). (d) Western blotting analysis of cyclin B1 expression levels in tumor lysates from indicated tumors treated with various concentrations of PEM (SCK, 0, 5, and 10%; CFPAC-1, 0, 1, and 5%).

Because the mammalian target of rapamycin complex 1 (mTORC1) signaling pathway is involved in the regulation of cell growth (e.g., amino acid translation, cell cycle, and angiogenesis) and tumorigenesis [20–22], we speculated whether the PEM inhibited mTORC1 activation status in tumor tissue. As expected, we found a decrease in phosphorylation of the mTORC1 targets p70S6K, ribosome protein S6, and eIF4E-binding protein 1 (4E-BP1) in tumor lysates from tumors implanted with the PEM compared with those in control tumors (Figures 2(a) and 2(b)). We also confirmed that total p70S6K and S6 levels were similar in PEM-implanted and control tumors (Figures 2(a) and 2(b)). As a result of the inhibition of mTORC1 by the PEM, both HIF-1*α* and HIF-1*β* protein levels decreased in PEM-implanted tumors (Figure 2(c)). Because activation of HIF-1*α* by mTOR is regulated through a direct interaction between HIF-1*α* and regulatory associated protein of mTOR (Raptor) [23], we determined whether the PEM blocked the interaction between Raptor and HIF-1*α in vivo*. We performed endogenous immunoprecipitation with anti-Raptor antibodies followed by the immunodetection with anti-HIF-1*α*. The results demonstrated that the PEM disrupted the Raptor-HIF-1*α* interaction in tumors in a dose-dependent manner (Figure 2(c)). As cyclin B1 is a regulatory subunit of mitosis-phase promoting factor, and its proper regulation is essential for the initiation of mitosis [24], we wondered whether cyclin B1 expression would be reduced by PEM implantation. As shown in Figure 2(d), cyclin B1 protein levels were markedly reduced in tumor lysates from PEM-implanted tumors. Downregulation of cyclin B1 might be responsible for mitotic arrest and inhibition of tumor growth by the PEM in the tumor-bearing mice. These results indicate that the PEM regulates the mitotic phase of the cell cycle, in part, by suppressing mTORC1 signaling.

### 3.3. The PEM Reduces CD31 and VEGFR2 Expression in Tumors

**Figure 3:**
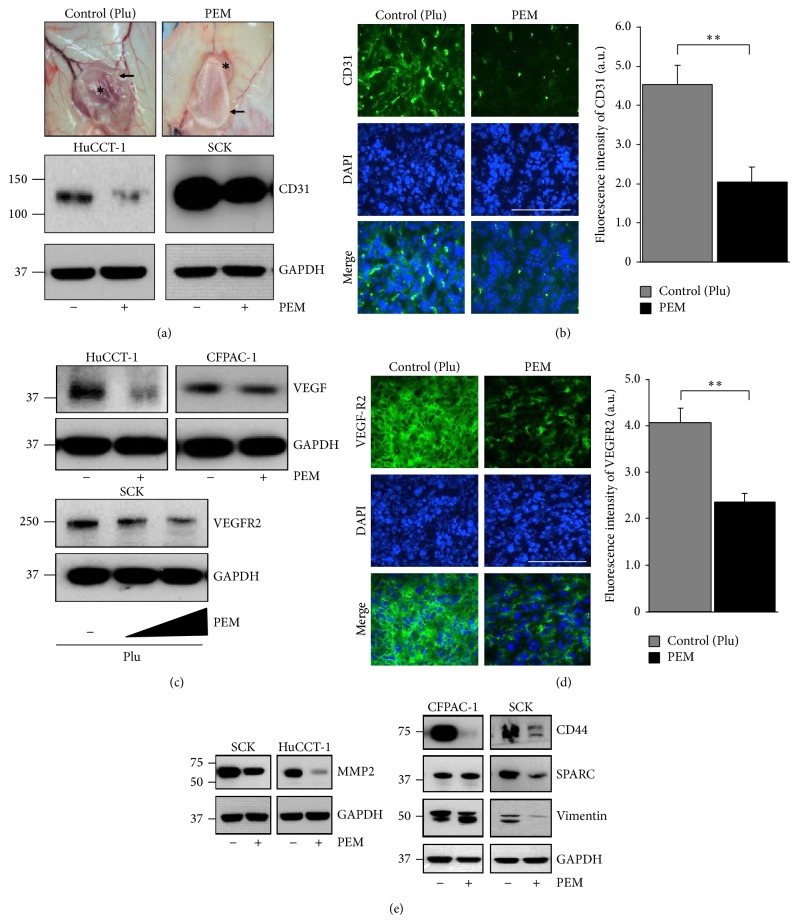
Paclitaxel-eluting membrane (PEM) implantation attenuates angiogenesis in xenografted tumors. (a) Representative photographs of the gross morphology of SCK tumors from nude mice implanted with the PEM or control (+Plu) membrane for 7 days. ∗, tumor; arrow, PEM; arrowhead, control (+Plu) membrane (upper). Western blot of CD31 expression in tumor lysates from indicated tumors implanted with the PEM or control. (b) Representative immunofluorescence analysis for CD31 in SCK tumors implanted with the PEM or control. Graph shows relative fluorescence intensity of CD31 (right). (c) PEM implantation decreased VEGF expression in tumor lysates from indicated tumors (upper). VEGFR2 expression was evaluated in SCK tumor tissue after implantation with 0, 5, and 10% paclitaxel in a PEM by immunoblotting (lower). (d) Representative immunofluorescence analysis for VEGFR2 in tissue from SCK tumors implanted with the PEM or control and relative fluorescence intensity of VEGFR2 (right). ((b), (d)) Positive protein staining is in green and nuclei are stained blue with DAPI. Original magnification, ×40. Scale bar: 50 *μ*m. Values are means ± standard deviation (SD). *N* = 9 fields per group. ^∗∗^ *P* < 0.01. (e) Effects of PEM implantation on the expression of MMP-2, CD44, SPARC, and vimentin proteins in indicated tumors. Proteins were detected by Western blotting using the indicated antibodies.

Because the tumor vasculature in xenograft animals is host-derived, we explored whether PEM implantation as a method for LDD had any role in vascularization. The gross assessment of tumors from mice treated with the PEM clearly showed a pale appearance with reduced vascularization and tumor volume. In contrast, control tumors appeared larger and well vascularized (Figure 3(a)). Tumor size and weight in animals treated with the PEM were significantly lower (4-5-fold) than those in tumors from controls (data not shown). To determine whether tumor shrinkage by the PEM corresponded to inhibited angiogenesis, we assessed microvascular density in the absence and presence of PEM-treated tumors by detecting the microvascular marker CD31 [25]. Figures 3(a) and 3(b) show that the PEM downregulated CD31 protein expression compared with that in control tumors. To address the signal transduction pathways by which angiogenesis was inhibited in the PEM-implanted tumors, VEGF protein levels were measured in tumors from control or PEM-treated mice by Western blotting. VEGF protein levels decreased in tumor lysates from PEM-implanted tumors (Figure 3(c)). Although VEGF protein levels decreased slightly, PEM implantation induced downregulation of the VEGFR2 protein in a dose-dependent manner in SCK tumors (Figure 3(c)). The immunofluorescence analysis confirmed that VEGFR2 protein expression decreased significantly in tumors from PEM-implanted mice, which was paralleled by reduced tumor vessel density (Figure 3(b)). Angiogenesis within the tumor microenvironment is a complex process regulated by pro- and antiangiogenic factors produced by both tumor cells and the stromal compartment [26]. We wondered whether PEM-mediated antiangiogenesis was caused by changes in VEGFR2 downstream signaling molecules. We found that PEM-implanted tumors exhibited decreased levels of matrix metalloproteinase 2 [27], an extracellular matrix (ECM) degrading component, compared with those in control tumors (Figure 3(e)). Expression of CD44 [28], which mediates cell-cell and cell-matrix interactions, secreted protein acidic and rich in cysteine (SPARC) [29], a stromal cell marker, and vimentin [30], a mesenchymal marker, decreased in PEM-implanted tumors relative to those in control tumors (Figure 3(e)). Taken together, these results suggest that decreasing tumor vasculature can limit the supply of nutrients and oxygen to tumor cells, thereby leading to antitumor effects. These findings indicate that the PEM inhibited angiogenesis by downregulating VEGF, VEGFR2, vascularization, and tumor-stromal interaction-related protein expression.

## 4. Discussion

We investigated protein profiles in whole tumors from pancreatic/cholangiocarcinoma xenografted tumor-bearing mice after PEM implantation to investigate the molecular mechanisms behind the paclitaxel-eluting stent for LDD and attenuation of tumor growth in a malignant biliary obstruction. Drug-eluting stent might ideally be used to treat the extrahepatic bile duct cancer that grows in the lining of the bile ducts. Unlike biliary cancer, a malignant biliary obstruction with pancreatic cancer, which occurs from extrinsic compression, lacks rationale for utilizing paclitaxel-eluting stent implants. However, MSCPM-II could be expected to have an antitumor effect by suppressing tumor in-growth from a combination of systemic or radiation treatment. Innovative strategies for local and prolonged delivery of approved chemotherapeutic drugs (e.g., paclitaxel) from MSCPM-II in patients with inoperable malignant biliary obstruction are currently under investigation. Local application of a chemotherapeutic agent in a stent can minimize the systemic side effects of the agent while maximizing its concentration within the bile duct. Here, we show that the PEM is a LDD device that supplies paclitaxel to tumors and inhibits their growth.

The hallmarks of cancers are deregulation of the cell cycle machinery, self-sufficiency of growth signals, insensitivity to growth inhibitory signals, evasion of apoptosis, tissue invasion, metastasis, and sustained angiogenesis [31]. Tumor progression requires angiogenesis, which is generally induced in response to hypoxia through a process known as the angiogenic switch [32–35]. In this study, we showed that the cancer signal transduction pathways were changed by the PEM implanted in pancreatic/cholangiocarcinoma xenografted tumors.

mTORC1 contains Raptor, which serves as a scaffolding protein for recruiting substrates for phosphorylation by the mTORC1kinase domain [36]. Activated mTORC1 regulates protein synthesis by directly phosphorylating S6K and 4E-BP1, which are translation-initiating factors important for cap-dependent mRNA translation and to increase the level of proteins needed for cell cycle progression, proliferation, and angiogenesis [37–39]. Notably, PEM implantation induced downregulation of mTORC1, dephosphorylated phospho-S6K, phospho-S6, and phospho-4E-BP1 and inhibited the protein synthesis required for angiogenesis and tumor growth.

Because tumor growth and metastasis are highly dependent on increased microvascular density, a reduction in the number of blood vessels is critical for antitumor responses. VEGF-VEGFR2 signal transduction leads to activation of various downstream signaling molecules responsible for endothelial cell migration, proliferation, and survival [25, 40]. Among the many changes in protein function that occur during tumor progression, alterations in cell-cell and cell-matrix adhesion seem to play a central role in facilitating tumor cell migration, invasion, and metastasis [41]. As expected, PEM implantation prevented tumor growth by affecting vascularization in both the pancreatic and cholangiocarcinoma models. We also observed that PEM implantation into xenograft tumors inhibited tumor growth by disrupting tumor invasion and metastatic behaviors, including the epithelial-to-mesenchymal transition and breakdown of the ECM. Therefore, the PEM induced inhibition of tumor growth in the xenografted tumor models might be explained by a combination of several mechanisms of action, such as antiproliferative, proapoptotic, and antiangiogenic effects.

The limitations of this study should be discussed. Because an orthotopic animal model of malignant biliary obstruction is not available so far, we should have investigated the molecular mechanism of the antitumor effect by the PEM as a LDD in subcutaneous xenografted nude mice. Although several strategies (e.g., fluorescent tagging and radioisotope labeling methods) have been developed to evaluate the distribution, penetration and distribution of a drug from a drug-eluting membrane into tumors remain a major challenge in cancer chemotherapy because distribution is impeded by several factors related to the physicochemical characteristics of the drug and the tumor tissue. Further investigations are needed to determine the mode of drug distribution from a drug-eluting membrane into tumors. Paclitaxel is suitable as a chemotherapeutic agent in a drug-eluting stent due to its lipophilic character and broad-spectrum anticancer effects, but gemcitabine is clinically preferred for treating a malignant biliary obstruction; thus, further improvements in hydrophilic agents (e.g., gemcitabine) for use in drug-eluting stents should be investigated.

## 5. Conclusion

We report that local delivery of paclitaxel from a PEM inhibited growth of inoculated pancreatic cancer and cholangiocarcinoma in nude mice by suppressing angiogenesis via the mTORC1/inducing apoptosis signaling pathway. The understanding of the molecular mechanisms behind the paclitaxel-eluting stent will change the paradigm for more successful treatment of patients with malignant biliary obstructions.

## Supplementary Material

The animal toxicity at each of these PEM was evaluated by weighing the mice. There were no deaths but some weight loss. Most of mice implanted with PEM experienced mild weight loss (<5%) during the 2 days after implantation. The mice began to regain weight 6 days after the PEM implantation. Therefore, based on observation of body weight no other host toxicities were observed.

## References

[1] Wagner H.-J., Knyrim K., Vakil N., Klose K. J. (1993). Plastic endoprostheses versus metal stents in the palliative treatment of malignant hilar biliary obstruction. A prospective and randomized trial. *Endoscopy*.

[2] Smith A. C., Dowsett J. F., Russell R. C. G., Hatfield A. R. W., Cotton P. B. (1994). Randomised trial of endoscopic stenting versus surgical bypass in malignant low bileduct obstruction. *The Lancet*.

[3] Lee D. K. (2009). Drug-eluting stent in malignant biliary obstruction. *Journal of Hepato-Biliary-Pancreatic Surgery*.

[4] Lee D. K., Kim H. S., Kim K.-S. (2005). The effect on porcine bile duct of a metallic stent covered with a paclitaxel-incorporated membrane. *Gastrointestinal Endoscopy*.

[5] Suk K. T., Kim J. W., Kim H. S. (2007). Human application of a metallic stent covered with a paclitaxel-incorporated membrane for malignant biliary obstruction: multicenter pilot study. *Gastrointestinal Endoscopy*.

[6] Jang S. I., Kim J. H., You J. W. (2013). Efficacy of a metallic stent covered with a paclitaxel-incorporated membrane versus a covered metal stent for malignant biliary obstruction: a prospective comparative study. *Digestive Diseases and Sciences*.

[7] Kalinowski M., Alfke H., Kleb B., Dürfeld F., Wagner H. J. (2002). Paclitaxel inhibits proliferation of cell lines responsible for metal stent obstruction: possible topical application in malignant bile duct obstructions. *Investigative Radiology*.

[8] Jang S. I., Kim J.-H., Kim M. (2012). Porcine feasibility and safety study of a new paclitaxel-eluting biliary stent with a Pluronic-containing membrane. *Endoscopy*.

[9] Jordan M. A., Wendell K., Gardiner S., Derry W. B., Copp H., Wilson L. (1996). Mitotic block induced in HeLa cells by low concentrations of paclitaxel (taxol) results in abnormal mitotic exit and apoptotic cell death. *Cancer Research*.

[10] Schiff P. B., Horwitz S. B. (1980). Taxol stabilizes microtubules in mouse fibroblast cells. *Proceedings of the National Academy of Sciences of the United States of America*.

[11] Belotti D., Vergani V., Drudis T. (1996). The microtubule-affecting drug paclitaxel has antiangiogenic activity. *Clinical Cancer Research*.

[12] Lau D. H., Xue L., Young L. J., Burke P. A., Cheung A. T. (1999). Paclitaxel (Taxol): an inhibitor of angiogenesis in a highly vascularized transgenic breast cancer. *Cancer Biotherapy and Radiopharmaceuticals*.

[13] Rowinsky E. K. (1997). Paclitaxel pharmacology and other tumor types. *Seminars in Oncology*.

[14] Skwarczynski M., Hayashi Y., Kiso Y. (2006). Paclitaxel prodrugs: toward smarter delivery of anticancer agents. *Journal of Medicinal Chemistry*.

[15] Liu R., Wolinsky J. B., Catalano P. J. (2012). Paclitaxel-eluting polymer film reduces locoregional recurrence and improves survival in a recurrent sarcoma model: a novel investigational therapy. *Annals of Surgical Oncology*.

[16] Gavathiotis E., Suzuki M., Davis M. L. (2008). BAX activation is initiated at a novel interaction site. *Nature*.

[17] Huang D. C., Strasser A. (2000). BH3-only proteins—essential initiators of apoptotic cell death. *Cell*.

[18] Liao P.-C., Tan S.-K., Lieu C.-H., Jung H.-K. (2008). Involvement of endoplasmic reticulum in paclitaxel-induced apoptosis. *Journal of Cellular Biochemistry*.

[19] Zinszner H., Kuroda M., Wang X. (1998). CHOP is implicated in programmed cell death in response to impaired function of the endoplasmic reticulum. *Genes and Development*.

[20] Fasolo A., Sessa C. (2008). mTOR inhibitors in the treatment of cancer. *Expert Opinion on Investigational Drugs*.

[21] Fingar D. C., Richardson C. J., Tee A. R., Cheatham L., Tsou C., Blenis J. (2004). mTOR controls cell cycle progression through its cell growth effectors S6K1 and 4E-BP1/eukaryotic translation initiation factor 4E. *Molecular and Cellular Biology*.

[22] Wolpin B. M., Hezel A. F., Abrams T. (2009). Oral mTOR inhibitor everolimus in patients with gemcitabine-refractory metastatic pancreatic cancer. *Journal of Clinical Oncology*.

[23] Land S. C., Tee A. R. (2007). Hypoxia-inducible factor 1*α* is regulated by the mammalian target of rapamycin (mTOR) via an mTOR signaling motif. *Journal of Biological Chemistry*.

[24] Yuan J., Yan R., Krämer A. (2004). Cyclin B1 depletion inhibits proliferation and induces apoptosis in human tumor cells. *Oncogene*.

[25] Senger D. R., Claffey K. P., Benes J. E., Perruzzi C. A., Sergiou A. P., Detmar M. (1997). Angiogenesis promoted by vascular endothelial growth factor: regulation through a1*β*1 and *α*2*β*1 integrins. *Proceedings of the National Academy of Sciences of the United States of America*.

[26] Hanahan D., Weinberg R. A. (2011). Hallmarks of cancer: the next generation. *Cell*.

[27] Ellenrieder V., Alber B., Lacher U. (2000). Role of MT-MMPs and MMP-2 in pancreatic cancer progression. *International Journal of Cancer*.

[28] He Y., Wu G. D., Sadahiro T. (2008). Interaction of CD44 and hyaluronic acid enhances biliary epithelial proliferation in cholestatic livers. *The American Journal of Physiology—Gastrointestinal and Liver Physiology*.

[29] Neuzillet C., Tijeras-Raballand A., Cros J., Faivre S., Hammel P., Raymond E. (2013). Stromal expression of SPARC in pancreatic adenocarcinoma. *Cancer and Metastasis Reviews*.

[30] Sato Y., Harada K., Itatsu K. (2010). Epithelial-mesenchymal transition induced by transforming growth factor-*β*1/snail activation aggravates invasive growth of cholangiocarcinoma. *The American Journal of Pathology*.

[31] Hanahan D., Weinberg R. A. (2000). The hallmarks of cancer. *Cell*.

[32] Balsari A., Maier J. A. M., Colnaghi M. I., Ménard S. (1999). Correlation between tumor vascularity, vascular endothelial growth factor production by tumor cells, serum vascular endothelial growth factor levels, and serum angiogenic activity in patients with breast carcinoma. *Laboratory Investigation*.

[33] Bergers G., Benjamin L. E. (2003). Tumorigenesis and the angiogenic switch. *Nature Reviews Cancer*.

[34] Folkman J., Shing Y. (1992). Angiogenesis. *Journal of Biological Chemistry*.

[35] Grant D. S., Kibbey M. C., Kinsella J. L., Cid M. C., Kleinman H. K. (1994). The role of basement membrane in angiogenesis and tumor growth. *Pathology Research and Practice*.

[36] Hara K., Maruki Y., Long X. (2002). Raptor, a binding partner of target of rapamycin (TOR), mediates TOR action. *Cell*.

[37] Guertin D. A., Sabatini D. M. (2005). An expanding role for mTOR in cancer. *Trends in Molecular Medicine*.

[38] Efeyan A., Sabatini D. M. (2010). MTOR and cancer: many loops in one pathway. *Current Opinion in Cell Biology*.

[39] Gibbons J. J., Abraham R. T., Yu K. (2009). Mammalian target of rapamycin: discovery of rapamycin reveals a signaling pathway important for normal and cancer cell growth. *Seminars in Oncology*.

[40] Lin S.-Z., Wei W.-T., Chen H. (2012). Antitumor activity of emodin against pancreatic cancer depends on its dual role: promotion of apoptosis and suppression of angiogenesis. *PLoS ONE*.

[41] Christofori G. (2006). New signals from the invasive front. *Nature*.

