# Supplementary material for: Molecular Mechanism of Local Drug Delivery with Paclitaxel-Eluting Membranes in Biliary and Pancreatic Cancer: New Application for an Old Drug
Source: Gastroenterol Res Pract. 2015 Apr 23;2015:568981. doi: 10.1155/2015/568981 (PMC4423024; doi:10.1155/2015/568981)
Supplement: Supplementary file 1 — The animal toxicity at each of these PEM was evaluated by weighing the mice. There were no deaths but some weight loss. Most of mice implanted with PEM experienced mild weight loss (<5%) during the 2 days after implantation. The mice began to regain weight 6 days after the PEM implantation. Therefore, based on observation of body weight no other host toxicities were observed. [file 568981.f1.pdf]

Supplementary Materials

Cell lines and antibodies

PANC-1 and CFPAC-1 cells were purchased from the ATCC (Manassas, VA, USA). HuCCT-1 and SCK cells were procured from the Health Science Research Resources Bank (Osaka, Japan) and Dr. Dae-Ghon Kim of Chonbuk National University Medical School and Hospital (Jeonju, Korea), respectively. All cell lines were maintained in a humidified incubator at 37°C with 5% CO_2.

Antibodies against S6K, phospho-S6K, S6, phospho-S6, 4EBP1, phospho-4EBP1, LC3B, cleaved caspase-3, CHOP, Bax, Bim, BCl-2, cyclin B1, PKM2, phospho-PKM2, HIF-1β, CD44, SPARC, vimentin, acetyl CoA carboxylase, phospho-acetyl CoA carboxylase, and GAPDH were obtained from Cell Signaling Technology. CD-31 and VEGF were purchased from Abcam (Cambridge, MA, USA). HIF-1α, VEFGR2/Flk-1, and MMP-2 were obtained from Santa Cruz Biotechnology.

Tumor xenograft and treatment

Female 6–8-week-old athymic nude mice were purchased from Orient Bio (Kyunggido, Korea) for subcutaneous xenografts. To establish the tumor xenograft model, 2×10^6 cells were suspended in 200-µl plain growth media (DMEM or RPMI-1640) and injected subcutaneously into spaces under the dorsal skin. Tumors were measured every other day using calipers, and their volumes were calculated by the following formula: 0.5 × length × width × depth. The animal's body weight was monitored every other day. When tumor volume reached 100 mm^3, the mice were anesthetized with a mixture of Zoletil (30 mg/kg) and Rompun (10 mg/kg) i.p., and the PEMs were surgically implanted underneath the tumors.

Supplementary Figure Legends

Supplementary Figure S1. The effect of implanting the paclitaxel-eluting membrane (PEM) on growth of SCK xenografts in nude mice. Mice were implanted with bare membrane (control), Plu only (control + Plu), paclitaxel (PTX) 5% plus Plu, or PTX 10% plus Plu membrane for 21 days. Data represent means of eight mice per group; error bars = standard deviation. *, $P < .05$; **, $P < .01$ vs. control (Plu only).

Supplementary Figure S2. The effect of implanting the paclitaxel-eluting membrane (PEM) on growth of HuCCT-1 xenografts in nude mice. Mice were implanted with a bare membrane (control), Plu only (control+ Plu), paclitaxel (PTX) 5% plus Plu, or PTX 10% plus Plu membrane for 33 days. Data represent means of eight mice per group; error bars, standard deviation. *, $P < .05$ vs. control (Plu only).

Supplementary Figure S3. Western blots of phospho-S6K and cleaved caspase-3 in the protein extract from an SCK xenograft tumor section (proximal and distal) implanted with the paclitaxel-eluting membrane (PEM) (paclitaxel 10% + Plu) for 15 days. P, proximal to PEM; D, distal to PEM. The sizes of the molecular weight markers (in kilodaltons) are indicated on the left.

Supplementary Figure S4. A simplified schematic diagram of the molecular mechanisms responsible for the anti-tumor properties of the paclitaxel-eluting membrane in pancreatic/biliary cancer xenografts.

Supplementary Figure S5. The PEM induced apoptosis in CFPAC-1 tumors. BCl-2, Bim, and Bax expression was detected in tumor lysates by Western blotting. Bax and Bim were immunoprecipitated (IP) with Bax.

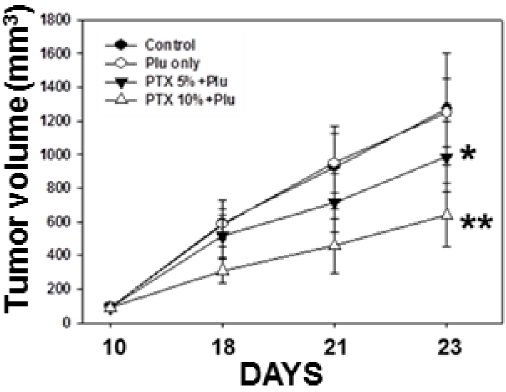

Supplementary Figure S1

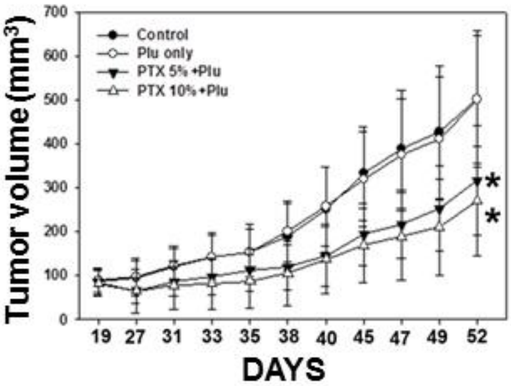

Supplementary Figure S2

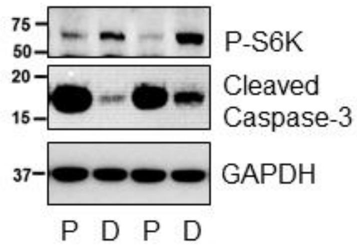

Supplementary Figure S3

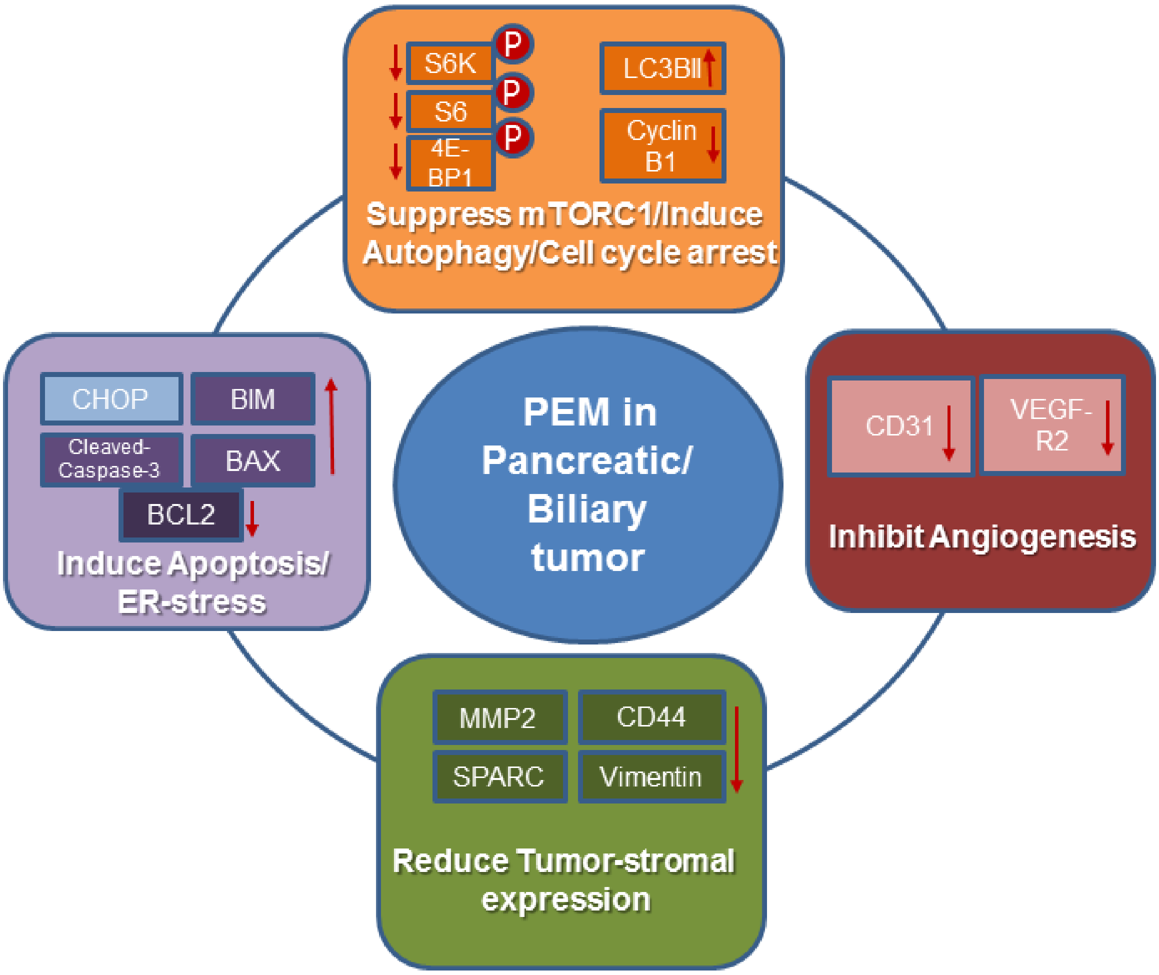

Supplementary Figure S4

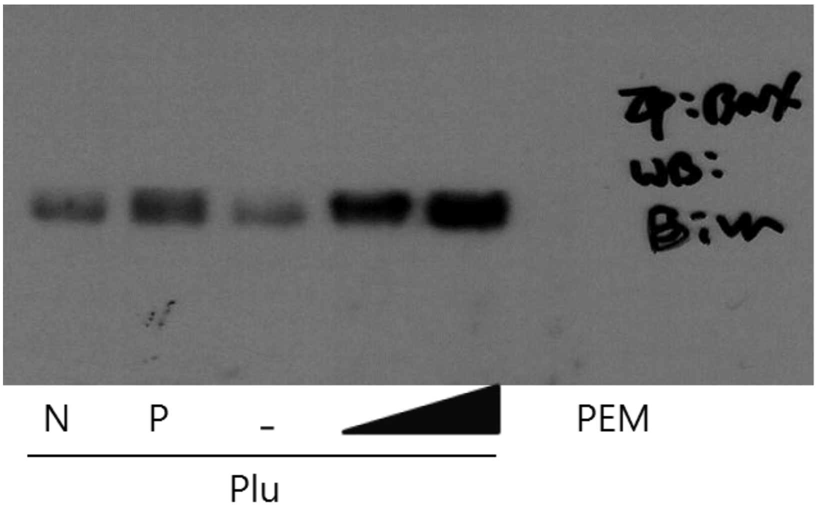

N: negative control
P: positive control

Supplementary Figure S5
